# Enhanced homing and efficacy of HER2-CAR T cells via CXCR5/CCR6 co-expression for HER2-positive NSCLC

**DOI:** 10.1186/s12967-025-06866-9

**Published:** 2025-08-05

**Authors:** Xiaoyuan Hu, Chunlei Ge, Caixiu Huang, Dan He, Xiaoxuan Yao, Jiaxing Cheng, Jiyin Guo, Ke Li, Yunshan Ye, Li Li, Jianchuan Xia, Tao Li, Hong Yao

**Affiliations:** 1 Cancer Biotherapy Center& Cancer Research Institute, The Third Affiliated Hospital of Kunming Medical University, Yunnan Cancer Hospital, Peking University Cancer Hospital Yunnan, Kunming, 650106 China; 2 State Key Laboratory of Oncology in South China, Guangdong Provincial Clinical Research Center for Cancer, Guangdong Key Laboratory of Nasopharyngeal Carcinoma Diagnosis and Therapy, Sun Yat-Sen University Cancer Center, Guangzhou, 510060 Guangdong People’s Republic of China

**Keywords:** Lung cancer, CAR T therapy, Tumor microenvironment, Specific cognate chemokine and receptor

## Abstract

**Background:**

Chimeric antigen receptor T-cell (CAR T) therapy development represents a promising therapeutic strategy for HER2-positive non-small cell lung cancer (NSCLC), a subtype accounting for 1–5% of NSCLC cases. However, the clinical efficacy of CAR T cells remains limited by poor tumor infiltration. Here, we identify NSCLC-specific overexpression of the CXCL13 and CCL20 chemokines within the tumor microenvironment (TME) and develop a dual chemokine receptor strategy to overcome this barrier.

**Methods:**

Western blotting and qRT-PCR were used to quantify chemokine receptor expression (CXCR5, CCR6) in NSCLC. Cytotoxicity and antigen recognition sensitivity of CXCR5-CCR6-HER2-CAR T cells against target cells were assessed using in vitro co-culture assays. In vitro proliferation and migration capacities of these engineered T cells were also evaluated. Anti-tumor activity was determined through in vivo animal experiments.

**Results:**

We demonstrate for the first time that HER2-targeted CAR T cells co-expressing the chemokine receptors CXCR5 and CCR6 selectively respond to CXCL13 and CCL20, which are highly expressed in the NSCLC TME. This dual chemokine receptor co-expression strategy has not been previously applied to solid tumors. The CXCR5/CCR6 pairing synergistically enhanced the antitumor activity of HER2-CAR T cells in both in vitro and in vivo models. Furthermore, CXCR5 and CCR6 co-expression significantly improved the in vitro cytotoxicity, antigen recognition sensitivity, proliferation, and migration of HER2-CAR T cells. In vivo, this modification enhanced HER2-CAR T cell survival, expansion, and tumor infiltration.

**Conclusion:**

CXCR5/CCR6 co-expression establishes a novel therapeutic paradigm for refractory HER2-positive NSCLC. Its modular design facilitates rapid clinical translation and adaptation to other chemokine-defined solid tumors.

**Supplementary Information:**

The online version contains supplementary material available at 10.1186/s12967-025-06866-9.

## Introduction

HER2-positive NSCLC, though a minority subtype (1–5%), presents distinct clinical challenges [1]. It is predominantly adenocarcinoma, exhibits high rates of central nervous system metastases, and demonstrates intrinsic resistance to conventional chemotherapy and EGFR inhibitors [2]. Unlike HER2-driven malignancies in other organs, no HER2-targeted therapies are currently approved for NSCLC, leaving patients with limited options and poor outcomes [3]. While antibody–drug conjugates (e.g., trastuzumab deruxtecan) show modest activity, they are limited by toxicity and acquired resistance [4]. CAR T cell therapy, which directly engages HER2-expressing tumor cells, presents a promising alternative.

Chimeric antigen receptor T (CAR T) cell therapy, a pioneering precision medicine technology, holds significant promise for addressing complex tumor treatment challenges [5]. Despite remarkable efficacy in hematological malignancies, CAR T application in solid tumors like NSCLC faces barriers including: lack of tumor-specific antigens, on-target/off-tumor effects, an immunosuppressive tumor microenvironment (TME), and limitations in T cell infiltration, homing, and exhaustion [6]. 

Chemokines within the TME exhibit functional dichotomy, with specific subsets driving opposing biological outcomes. Pro-tumorigenic chemokines (e.g., G-CSF, CCL2) promote cancer progression by recruiting immunosuppressive cells (TAMs, MDSCs, Tregs), inducing angiogenesis via VEGF activation, and facilitating metastasis through EMT induction and pre-metastatic niche formation [7, 8]. Conversely, anti-tumor chemokines (e.g., CXCL9, CXCL10, CCL5) mediate protective immunity by recruiting effector lymphocytes (CD8 + T cells, NK cells) while enhancing immune synapse formation and antigen presentation [9, 10]. This balance between pro- and anti-tumor chemokine networks is a strategic focus in cancer immunotherapy [11]. 

Clinical strategies targeting chemokines—depleting pro-tumor factors, administering anti-tumor chemokines, or engineering CAR T cells to express specific chemokine ligands/receptors—show considerable therapeutic promise [12–14]. Chemokine receptor engineering in CAR T cells has established precedents: CXCR4-engineered CAR T cells demonstrated enhanced migration toward CXCL12 gradients in ovarian cancer [15], while CXCR2-engineered CAR T cells improved trafficking in response to CXCL5 in glioblastoma [16]. These studies validate chemokine receptor engineering as a strategy to augment CAR T cell homing in solid tumors.

Unlike previous studies that primarily focused on engineering CAR T cells to express a single chemokine receptor, our study pioneers a dual chemokine receptor strategy by co-expressing CXCR5 and CCR6 in HER2-CAR T cells. We utilized the high-affinity orphan chemokine receptor-ligand mechanism, and selected the CXCL13/CXCR5 and CCL20/CCR6 combinations based on the specificity and complementary functions of the ligands and receptors [17, 18]. By analyzing the bioinformatics database and verifying the cancerous and non-cancerous tissues of different patients, we found a significant upregulation of CXCL13 and CCL20 in NSCLC versus normal lung tissue, establishing a tumor-directed chemokine gradient. The high specificity of CXCR5 and CCR6 for their cognate ligands minimizes off-target risks while exploiting their functional roles in recruiting and activating tumor-specific T cells within the TME [19, 20]. By co-expressing CXCR5 and CCR6, we engineered HER2-CAR T cells with enhanced homing capacity, specifically addressing the unmet therapeutic need in this refractory NSCLC subgroup.

## Materials and methods

### Cell lines

HEK 293 T, Jurkat, BEAS-2B, PC-9, A549, H1734, H1975, H1299, and HCC827 cells (Pricella, Wuhan, China) were cultured in Dulbecco’s Modified Eagle Medium or RPMI 1640 (HyClone) supplemented with 10% fetal bovine serum (HyClone). For xenograft modeling, HCC827 cells were stably transduced with green fluorescent protein and firefly luciferase fusion genes to establish HCC827-Luc/GFP cells.

### Patients sample

Lung adenocarcinoma (LUAD) tumor specimens were obtained from Yunnan Cancer Hospital (Kunming, China) with approval from the Institutional Review Board (KYCS2022186). All participants provided written informed consent. The study adhered to the Declaration of Helsinki guidelines.

### Chemokine expression analysis

The GEPIA tool [21], The Cancer Genome Atlas (TCGA), and Genotype-Tissue Expression (GTEx) database were used to analyze HER2, CXCL13, CCL20, CCL25, CX3CL1, CXCL12, and CXCL16 expression in LUAD versus normal lung tissues. We further assessed associations between CXCL13/CCL20 expression and LUAD patient overall survival were also evaluated.

### Cell line and tissue analysis

BEAS-2B, PC-9, A549, H1734, H1975, H1299, and HCC827 (well-characterized LUAD models) and six paired LUAD/adjacent tissues were analyzed. Protein and mRNA levels of HER2, CXCL13 and CCL20 were measured in whole-cell lysates of NSCLC cell lines and clinical tissue samples as previously described [22]. Lung adenocarcinoma cell lines and BEAS-2B cell lines were cultured in serum-free medium for 48 h. Then, ELISA (eBioscience, China) was used to detect the expressions of chemokines CXCL13 and CCL20 in the supernatant. HER2 surface expression on HCC827/BEAS-2B cells was assessed by flow cytometry using anti-HER2 antibody (BioLegend, #324908).

### Western blot analysis

Proteins were extracted with RIPA buffer (Beyotime, China, #P0013B) containing protease inhibitors. Protein concentration was determined by BCA assay (Thermo Fisher, #23225). Samples (20–30 µg) were separated by SDS-PAGE on 10% or 12% gels (Epizyme, China, #PG112/PG113) using a molecular weight marker (Epizyme, #WJ103) and transferred to PVDF membranes (Millipore, #IPVH00010). Membranes were probed with: rabbit anti-HER2 (Abcam, ab134182), anti-CXCL13 (Abcam, ab272874), anti-CCL20 (Cell Signaling, #769245), and HRP-conjugated goat anti-rabbit IgG (Proteintech, SA00001-2).

### Quantitative PCR

Genomic DNA was extracted from mouse tumors using a DNeasy Blood & Tissue Kit (TIANGEN, China). HER2, CXCL13, and CCL20 copy numbers were quantified on an ABI 7500 system using specific Scorpion primers/probes and 100 ng genomic DNA per reaction, as described. Standard curves were generated from template plasmids. Copy numbers were calculated using GraphPad Prism (La Jolla, CA, USA).

### CAR design and synthesis

The HER2-CAR single-chain variable fragment (scFv) was derived from the HER2 monoclonal antibody (FRP5-antibody), with variable heavy (VH) and light (VL) domains connected by a triple-repeat glycine-serine linker ((G4S)3) [23]. The carboxy-terminus was sequentially fused to the CD8α hinge/transmembrane domain, CD137 (4-1BB) and CD28 co-stimulatory motifs, and CD3ζ signaling domain. This sequence was synthesized by Sangon Biotech and cloned into the pCDH lentiviral vector downstream of the CMV promoter at the multicloning site (MCS), generating pCDH-HER2-CAR (Fig. S1). For HER2-CXCR5-CCR6-CAR, CXCR5 and CCR6 full-length sequences (separated by a T2A element) were synthesized and inserted into the MCS downstream of the EF1α promoter in pCDH-HER2-CAR, yielding pCDH-HER2-CXCR5-CCR6-CAR. All constructs were sequence-verified by Sangon Biotech (Shanghai).

### Determine the transduction efficiency of CAR lentivirus in T cells in vitro

To detect the positive rate of CAR in different groups of lentivirus transduction T cells in vitro, anti-human G4S-PE antibody (#38907, Cell Signaling Technology (CST), USA) was used to detect the expression of HER2-CARs on the cell surface. The expressions of CXCR5 and CCR6 on the cell surface were detected by anti-human-CXCR5-PerCp-Cy7 (#562781, BD Biosciences, USA), anti-human-CCR6-PE-Cy7 antiboday (#560620, BD Biosciences, USA). HER2-CAR + T cells were isolated by FACS sorting using anti-G4S antibodies. Data were acquired on a CytoFLEX Flow Cytometer (Beckman Coulter) and analyzed using CytExpert software or FlowJo version 7.6.1 (Tree Star).

### CD69 activation assay

According to previous study methods [24], Jurkat cells (1 × 10^5^) were co-cultured 18 h with different numbers of tumor cells (1 × 10^5^, 5 × 10^4^, 1 × 10^4^, 5 × 10^3^, 1 × 10^3^) in complete RPMI-1640 media + IL-2 (30 ng/mL). Cells were then washed with PBS, stained with anti-CD69 antibody (BioLegend, #310906) at 4 °C for 30 min, washed again, and analyzed by flow cytometry.

### Cytotoxicity and cytokine release assays in vitro

Cytotoxicity of Control-T (non-transduced), HER2-CAR T, HER2-CXCR5-CAR T, HER2-CCR6-CAR T, and HER2-CXCR5-CCR6-CAR T cells was assessed by co-culture assay [13]. Target cell lysis was quantified using a lactate dehydrogenase (LDH) release kit (Promega) after 24 h co-culture at E:T ratios (1:1 to 20:1). Supernatants from 24 h co-cultures were analyzed for IL-2, IFN-γ, and TNF-α via ELISA (eBioscience, China).

### Cell proliferation assay

Control-T, HER-CAR T, and HER2-CXCR5-CCR6-CAR T cells (5 × 10^3^ cells/100 µL) were co-cultured with target cells at 1:1 E/T ratios in transparent 96-well plates for 24 or 48 h. Subsequently, 10 µL of Cell Counting Kit-8 (Yeasen Biotech, Shanghai, China) was added and vortex-mixed per established protocol [12]. Following incubation at 37 °C with 5% CO_2_ for 2–6 h, absorbance was measured at 450 nm using a microplate reader to compare CAR T-cell proliferation across groups.

### Transwell migration assay

Migration efficiency of HER2-CAR T, HER2-CXCR5-CAR T, HER2-CCR6-CAR T, and HER2-CXCR5-CCR6-CAR T cells was assessed using Transwell chambers [14]. Complete T-cell medium contained RPMI-1640 (Thermo Fisher Scientific) supplemented with 10% fetal bovine serum (Biological Industries), 1% penicillin/streptomycin (Thermo Fisher Scientific), and 30 ng/mL recombinant human IL-2 (PeproTech). HER2-CAR T or HER2-CXCR5-CCR6-CAR T cells (1 × 10^5^ cells in 100 µL medium) were seeded in the upper chamber of 24-well inserts (8 μm pore size, Corning). Lower chambers contained either tumor cells or 0–200 ng/mL recombinant chemokines (CXCL13, CCL20; YEASEN). Studies have shown that the concentration range of the chemokines used is between 0 and 200 ng/mL, which is within the normal physiological range of the tumor microenvironment in solid tumors [25, 26]. After incubation at 37 ℃ for 4–12 h, the CAR T cells that migrated to the lower chamber were absolutely counted using a flow cytometer (Beckman CytoFLEX LX). Specific migration was calculated as: Migration (%) = (Number of migrated cells in lower chamber/1 × 10^5^ total seeded cells) × 100. Background migration (negative control: no chemokines/tumor cells) was subtracted from all groups.

### Xenograft tumor model and in vivo evaluation

Female NSG mice (NOD.Cg-Prkdcscid Il2rgtm1Wjl/SzJ; 4–8 weeks) from Vital River Technology (Beijing, China) were housed under specific pathogen-free conditions at Yunnan University. All animal studies employed randomization and blinding. HCC827-Luc cells (1 × 10⁶) in 50 µL suspension were injected into the left lung parenchyma via thoracic incision following anesthesia with sodium pentobarbital (50 mg/kg). Mice (n = 5/group) received intravenous tail vein injections of 1 × 10⁷ T cells (100 µL; Control-T, HER2-CAR T, HER2-CXCR5-CAR T, HER2-CCR6-CAR T, or HER2-CXCR5-CCR6-CAR T) on days 14 and 28. This dosing regimen was selected based on established efficacy/safety profiles for HER2-targeted CAR T cells in murine solid tumors [27–29]. The initial infusion (Day 14) coincided with confirmed tumor engraftment (bioluminescence ≥ 80,000 p/s), while the second (Day 28) addressed anticipated T-cell contraction and tumor regrowth [30]. Safety was monitored via weekly body weight measurements. Tumor progression was tracked by weekly bioluminescence imaging after intraperitoneal anesthesia with tribromoethanol (300 mg/kg) and tail vein injection of D-luciferin (150 mg/kg; Promega). Images were acquired 3 min post-injection using an IVIS Spectrum system (PerkinElmer; 1-min exposure) and quantified with Living Image software (PerkinElmer). The study concluded when non-treated mice became moribund.

### In vivo migration assessment

Peripheral blood T-cell infiltration was analyzed by flow cytometry on days 21 and 42. Anti-human CD3-PE (#980008, BioLegend, American), CD4-APC (#980812, BioLegend, American), CD8-FITC (#98090, BioLegend, American) were used to detect the expressions of CD3-T, CD4-T and CD8-T cells in the peripheral blood. The expressions of HER2, CXCR5 and CCR6 on the cell surface of different CAR-T cell groups in peripheral blood were detected by using Anti-human CD3-PE, HER-2-PerCP (#324416, BioLegend, American), anti-human-CXCR5-PerCp-Cy7 and anti-human-CCR6-PE-Cy7 antiboday.

Tumors were excised and weighed at endpoint (Day 42). Single-cell suspensions from digested tumors (Mouse Tumor Dissociation Kit, Miltenyi Biotec #130-096-730) underwent Percoll gradient centrifugation. Flow cytometry with Anti-human CD3-P, CD4-AP, CD8-FITC, PD-1-APC (#379208, BioLegend, USA), and HER-2-PerCP antibodies quantified tumor-infiltrating CAR T cells, CD4 +/CD8 + CAR T subsets, and PD-1 + CAR T cells. At endpoint (day 42), CAR copy numbers in tumors were determined by RT-PCR. For immunohistochemistry (IHC), tumor specimens were fixed in 4% paraformaldehyde and stained with anti-CD4 (EPR685, abcam) and CD8α (CAL66, abcam; Servicebio, Wuhan, China). Positive staining regions were quantified using ImageJ.

### Histopathological analysis

Heart and liver tissues harvested at Day 42 were fixed in 10% neutral buffered formalin (48 h) and paraffin-embedded. 5-μm sections were H&E-stained following standard protocols.

## Results

### Chemokine expression in NSCLC

**Fig. 1:**
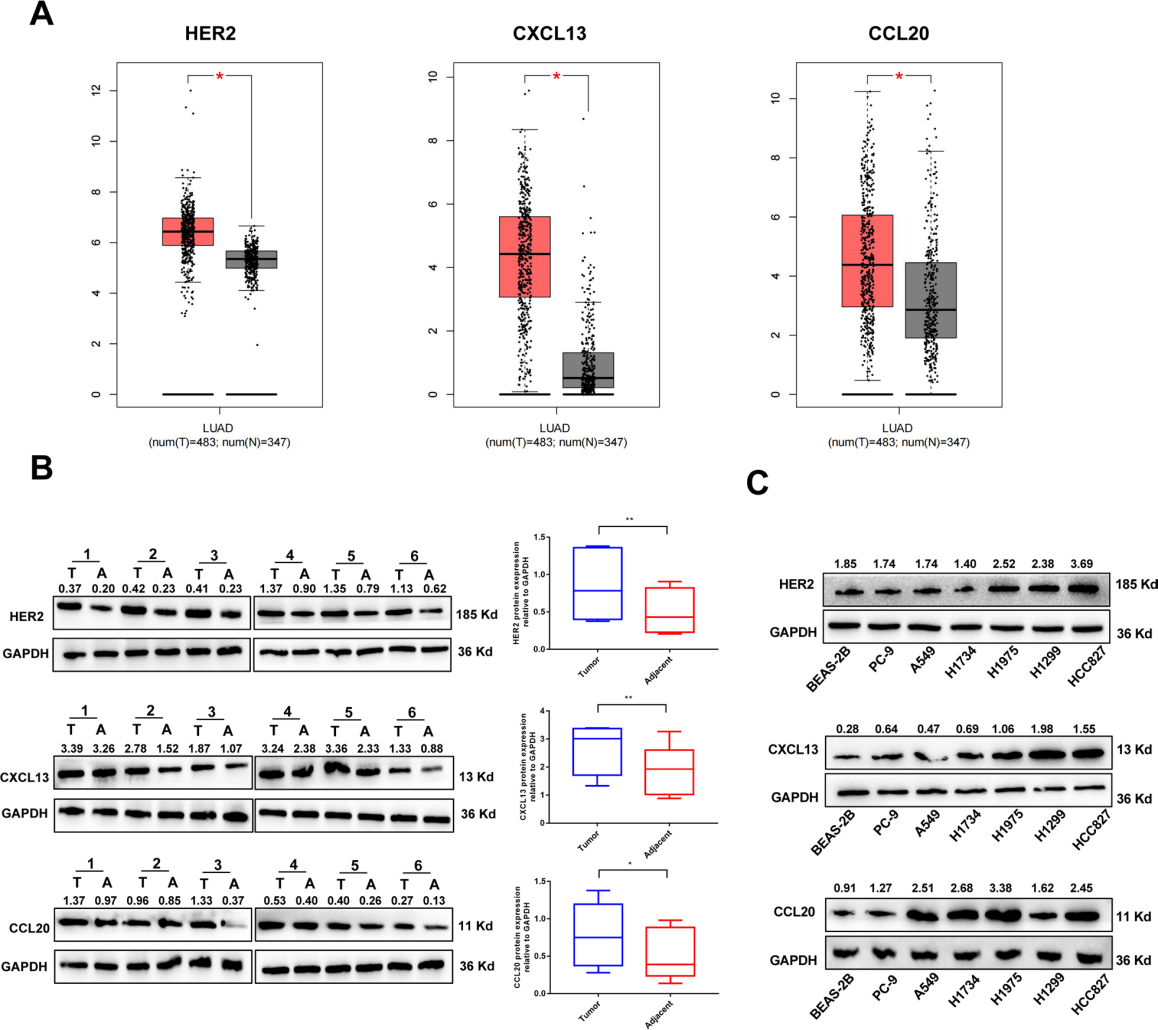
HER2 and chemokine CXCL13 and CCL20 expression profiles in LUAD tumors and cell lines. **A** Expression of HER2, CXCL13, and CCL20 in patients with lung adenocarcinoma (LUAD) determined using the online tool GEPIA. **B**, **C** Western blot analysis of HER2, CXCL13, and CCL20 expression in cellular lysates from: **B** Paired LUAD tumors (T) vs. adjacent normal tissues (N); **C** NSCLC cell lines vs. normal bronchial epithelium (BEAS-2B). Data represent the mean ± standard error of the mean (SEM) of three independent experiments. Welch’s t-test, paired t-test and Two-way ANOVA was conducted for statistical analysis. *P < 0.05, **P < 0.01

NSCLC exhibits the high expression of the chemokines CXCL13 and CCL20. Based on a recent review by Kobold et al. [31], we selected six orphan chemokine receptor–ligand pairs for further investigation due to their high specificity: CX3CL1–CX3CR1, CXCL16–CXCR6, CXCL13–CXCR5, CXCL12–CXCR4, CCL25–CCR9, and CCL20–CCR6 (Fig. S2A). To assess the relevance of these chemokines in lung cancer, we analyzed their expression profiles in 483 lung adenocarcinoma samples and 347 normal tissue samples from the TCGA and GTEx databases. CXCL13 and CCL20 were significantly overexpressed in cancerous tissues compared to normal lung tissues, while the expression of other chemokines showed no significant differences or exhibited opposite trends (e.g., CCL25, CX3CL1, CXCL16, and CXCL12) (Fig. 1A and Fig. S2B). We used TCGA and GTEx database to analyze the relationship between the expression levels of CXCL13 and CCL20 and the overall survival rate in patients with lung adenocarcinoma (LUAD). The results showed that the high expression of CCL20 was significantly associated with the reduction of the overall survival rate in patients with LUAD (log-rank *P* = 0.021). The high expression of CXCL13 was also not significantly correlated with the overall survival rate (log-rank *P* = 0.32) (Fig. S2C).

We further evaluated the expression of HER2 and found that HER2 is highly expressed in lung adenocarcinoma tissues based on data from the TCGA and GTEx databases (Fig. 1A). To validate these findings, we analyzed the transcriptional and protein expression levels of HER2, CXCL13, and CCL20 in six pairs of clinical lung adenocarcinoma tissue samples. At both the transcriptional and protein levels of HER2, CXCL13 and CCL20 expression was significantly elevated in cancerous tissues compared to that in adjacent non-cancerous tissues (Fig. 1B and Fig. S3).

Western blotting and qRT-PCR analyses were performed to quantify the expression levels of HER2, CXCL13, and CCL20 in the normal bronchial epithelial cell line BEAS-2B and several lung cancer cell lines (PC9, A549, H1734, H1975, H1299, and HCC827). Compared to BEAS-2B, HER2 protein expression was more than twice as high in HCC827 (Fig. 1C, Fig. S4A and Table. S1). Notably, all lung cancer cell lines exhibited elevated protein expression of CXCL13 and CCL20, with CXCL13 being approximately three times higher in H1975, H1299, and HCC827, and CCL20 being more than twice as high in A549, H1734, H1975, and HCC827. Both H1975 and HCC827 demonstrated the highest protein expression of CXCL13 and CCL20 (Fig. 1C). Similarly, the cell culture supernatant also had the highest levels of CXCL13 and CCL20 secreted by the HCC827 cell line (Fig. S4B). QRT-PCR results also showed that HCC827 cell line had higher mRNA levels of HER2, CXCL13 and CCL20 (Fig. S4C and Table. S2). Flow cytometry (FC) analysis revealed that HCC827 cells exhibited significantly higher surface expression of HER2 compared to BEAS-2B cells. The expression level of HER2 on HCC827 cells was approximately 10 times that on BEAS-2B cells, indicating that HER2 was strongly expressed on the surface of HCC827 cells (Fig. S4D). These findings suggest that the TME in lung adenocarcinoma is characterized by high levels of CXCL13 and CCL20. Given that the HCC827 cell line exhibited the highest levels of HER2, CXCL13, and CCL20, it was selected as the target cell line for subsequent experiments.

### Co-expression of CXCR5 and CCR6 enhanced the antigen recognition sensitivity and cytotoxic activity of HER2-CAR T cells in vitro

**Fig. 2:**
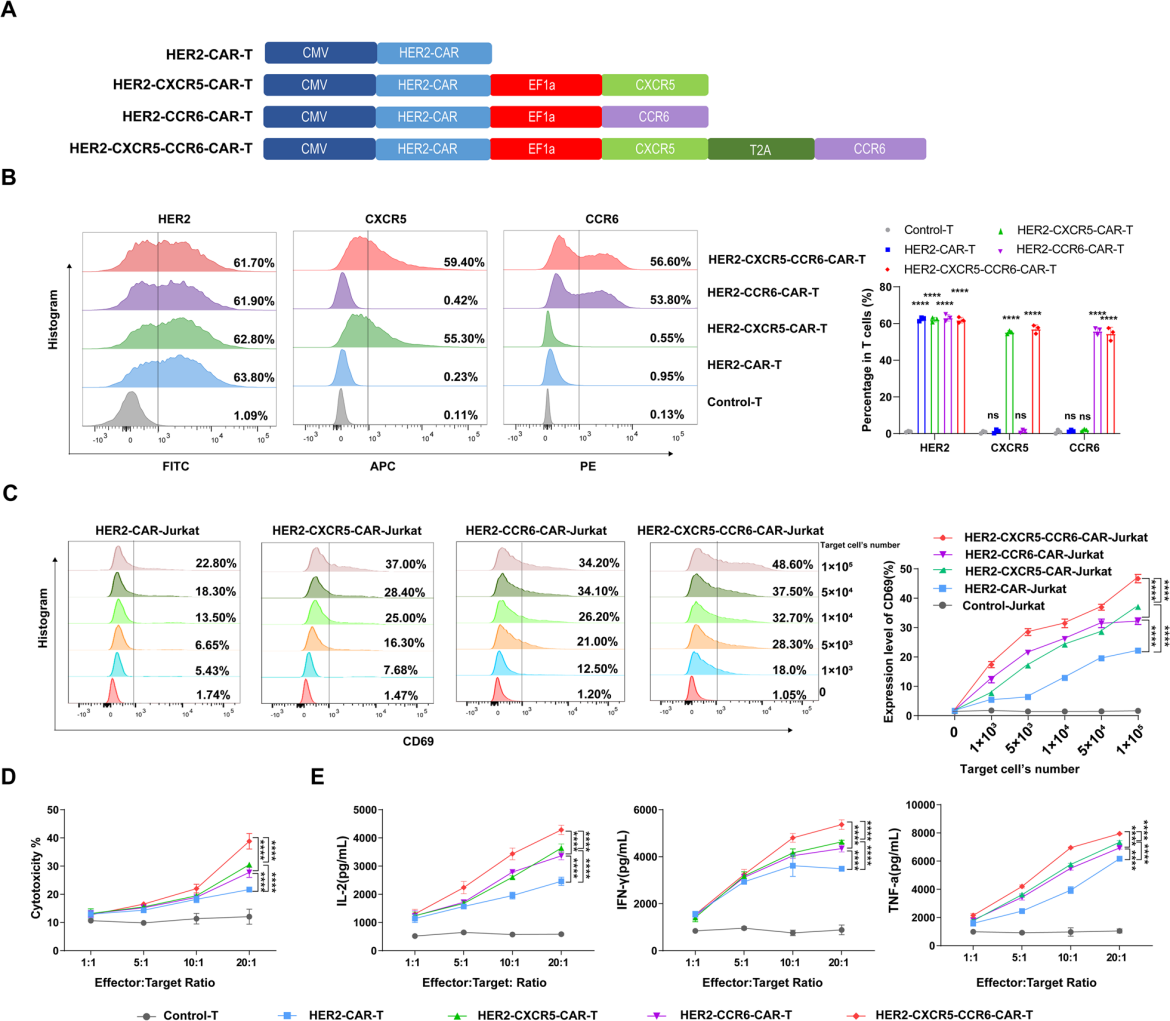
CXCR5 and CCR6 promote the cytotoxicity activity of HER2-CAR T cells in vitro. **A** Schematic of the modular composition of HER2-targeted conventional and cytokine CARs. **B** Expression of transgenes in lentivirus-transduced T cells analyzed by flow cytometry using the HER2-protein, anti-CXCR5 antibody, and anti-CCR6 antibody. **C** Co-culture Control-, HER2-CAR-, HER2-CXCR5-CAR-, HER2-CCR6-CAR-, or HER2-CXCR5-CCR6-CAR-Jurkat cells with varying numbers (0, 1 × 10^3^, 5 × 10^3^, 1 × 10^4^, 5 × 10^4^, 1 × 10^5^) of HCC827 cells after 18 h, after which the expression of CD69 in Jurkat cells was assessed using flow cytometry. **D** Cytotoxicity assay in the HCC827 cell line targeted by Control-, HER2-CAR-, HER2-CAR-CXCR5-, HER2-CAR-CCR6-, or HER2-CAR-CXCR5-CCR6-CAR Ts at E/T ratios from 1:1 to 20:1, determined by a lactate dehydrogenase assay. **E** Different CAR T cells were co-cultured with HCC827 cells at E/T ratios from 1:1 to 20:1; after 24 h, the production of IL-2, IFN-γ, and TNF-α by T cells was determined by ELISA. Data represent the mean ± SEM of three independent experiments. Unpaired t-test and Two-way ANOVA was conducted for statistical analysis. ****P < 0.0001, ns, no statistical significance

Leveraging our finding of high HER2, CXCL13, and CCL20 expression in the NSCLC TME (Fig. 1), we designed HER2-CAR T cells that either singly expressed the orphan chemokine receptors CXCR5 or CCR6 or co-expressed both high-specificity orphan pairing receptors CXCR5 and CCR6 (Fig. 2A). Flow cytometry analyses revealed that all the CAR T cell variants (HER2-CXCR5-CCR6-CAR T, HER2-CXCR5-CAR T, and HER2-CCR6-CAR T) exhibited a HER2 CAR expression positivity rate exceeding 60% (Fig. 2B). In both the co-expressing and singly expressing chemokine receptor CAR T cells, the expression rates of CXCR5 and CCR6 approached 60% (Fig. 2B). We sorted and purified the highly positive HER2-CAR T cells using FACS after transfection (Fig. 4E).

To assess the sensitivity and specificity of the CARs, we employed a high-throughput method developed by Bloemberg for evaluating CAR recognition in Jurkat cells [32]. As target cell density increased in the HCC827 cells, we observed a corresponding rise in CD69 expression among all the CAR-Jurkat cell lines, indicating the specific recognition of the HER2 antigen (Fig. 2C and Fig. S5A, B). Notably, under identical antigen density conditions, CD69 expression was significantly higher in HER2-CXCR5-CCR6-CAR-Jurkat cells compared to that in the singly-expressing CXCR5 or CCR6 CAR-Jurkat cells, with HER2-CAR-Jurkat cells showing the lowest CD69 levels (Fig. 2C and Fig. S5 A, B). When the target cells were presented with 1 × 10^5^ cells, CD69 expression in the co-expressing HER2-CXCR5-CCR6-CAR-Jurkat cells peaked at nearly 48.6%, while the singly-expressing CXCR5 and CCR6 groups showed lower levels of 37.0% and 34.2%, respectively. The HER2-CAR-Jurkat cells exhibited the lowest CD69 expression at 22.8% (Fig. 2C, Fig. S5 A, B).

Further experiments confirmed that increasing the effector-to-target (E:T) ratio enhanced the cytotoxicity against HCC827 target cells, as well as the secretion of the pro-inflammatory cytokines IL-2, IFN-γ, and TNF-α across all the CAR T cell groups (Fig. 2D, E and Table. S3). At identical and elevated E:T ratios, the HER2-CXCR5-CCR6-CAR T cells demonstrated the highest cytotoxic activity and cytokine secretion, surpassing the singly-expressing CXCR5 or CCR6 groups. The HER2-CAR T cells exhibited the lowest activity in these assays (Fig. 2D, E and Table. S3). These results suggest that the co-expression of CXCR5 and CCR6 significantly enhances both the antigen recognition sensitivity and cytotoxic efficacy of HER2-CAR T cells, highlighting the potential of this dual-receptor approach for improving CAR T cell therapy in solid tumors.

### Co-expression of CXCR5 and CCR6 enhances the proliferation and migration abilities of HER2 CAR T cells

**Fig. 3:**
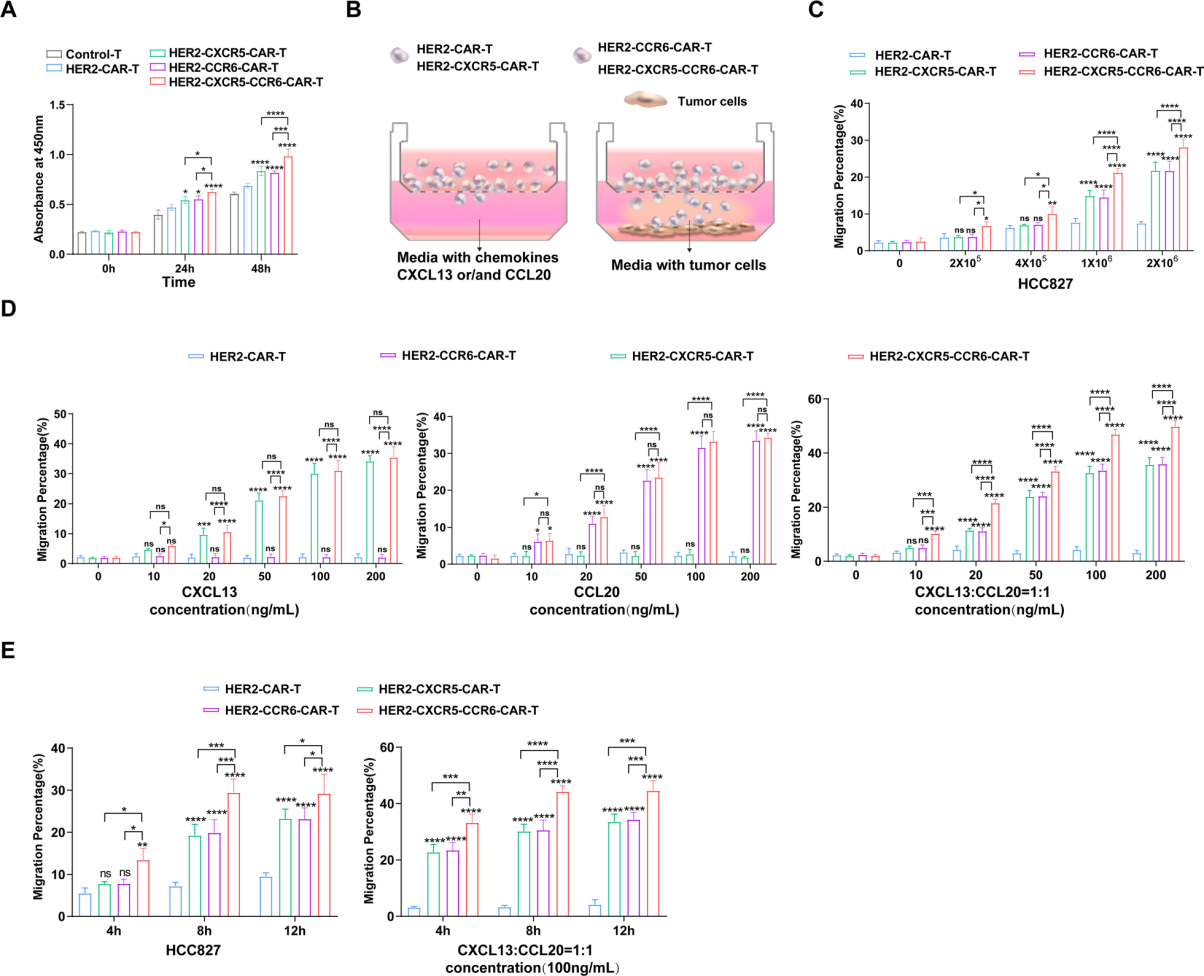
CXCR5 and CCR6 promote the migration and proliferation of HER2-CAR T cells in vitro*.* **A** Control-, HER2-CAR-, HER2-CXCR5-CAR-, HER2-CCR6-CAR-, or HER2-CXCR5-CCR6-CAR T cells were co-cultured with HCC827 cells at an E/T ratio of 1:1 for 0 h, 24 h, and 48 h. After co-culturing, the Cell Counting Kit-8 assay was used to assess the proliferation of T cells in different groups. **B** Graphical representation of the Transwell assay. **C**, **D** After incubation for 8 h with different numbers of HCC827 cells (**C**) or with different doses of chemokines (**D**), the migrated cells in the lower chamber were counted by flow cytometry, and the specific migration percentage was calculated according to the formula shown in the Materials and Methods section. **E** After incubation for 4, 8, and 12 h with 2 × 10^6^ HCC827 cells or with 100 ng/mL chemokines CXCL13 and CCL20, the migrated cells in the lower chamber were counted. The specific migration percentage was calculated according to the formula shown in the Materials and Methods section. Data represent the mean ± SEM of three independent experiments. Two-way ANOVA was conducted for statistical analysis. *P < 0.05, **P < 0.01, ***P < 0.001, ****P < 0.0001, ns, no statistical significance

To evaluate the effects of co-expressing or singly expressing CXCR5 and CCR6 on the functionality of HER2 CAR T cells, we first assessed their proliferative capacity. Under equal stimulation with HCC827 target cells, the proliferation of HER2-CXCR5-CCR6-CAR T cells was significantly higher compared to that of HER2-CAR T cells expressing either CXCR5 or CCR6 alone. The HER2-CAR T cells exhibited the lowest proliferative response following antigen stimulation (Fig. 3A).

Next, we employed Transwell assays to assess the migratory and chemotactic behaviors of the CAR T cells, either co-expressing or singly expressing CXCR5 and CCR6, in response to HCC827 target cells and chemokine gradients (Fig. 3B). As the number of HCC827 target cells increased, the chemotactic response of HER2-CXCR5-CCR6-CAR T cells progressively improved. In contrast, CXCR5- or CCR6-single-expressing T cells exhibited significant chemotaxis only at higher antigen densities, whereas HER2-CAR T cells showed negligible migratory responses (Fig. 3C).

To confirm that the chemotactic effects of the HER2-CXCR5-CCR6-CAR T cells were driven by the chemokines CXCL13 and CCL20 secreted by the HCC827 target cells, we performed gradient chemokine stimulation assays (Fig. 3D). The chemotactic migration of the HER2-CXCR5-CCR6-CAR T cells increased with the concentration of the chemokine stimulation. This effect was observed whether CXCL13 or CCL20 alone, or a 1:1 mixture of both, was used. At lower concentrations (0–10 ng/mL), the chemotactic response of the HER2-CXCR5-CCR6-CAR T cells to the mixture of CXCL13 and CCL20 was significantly stronger than to either chemokine alone. Although both chemokines promoted chemotaxis to a similar extent, the combined chemokine stimulation at low concentrations enhanced migration more effectively than did single chemokine exposure (Fig. 3D). The CAR T cells expressing CXCR5 or CCR6 alone showed specific chemotaxis toward the corresponding chemokine in the lower chamber (20 ng/mL), while the HER2-CAR T cells exhibited virtually no chemotactic effect (Fig. 3D).

Further analysis showed that the chemotactic effect of the HER2-CXCR5-CCR6-CAR T cells increased progressively with prolonged stimulation by either HCC827 target cells or a mixture of CXCL13 and CCL20. Notably, the co-expression group exhibited a significantly stronger chemotactic response over time compared to the single-expression groups, whereas the HER2-CAR T cells showed minimal chemotactic activity despite extended stimulation (Fig. 3E). These findings demonstrate that the co-expression of CXCR5 and CCR6 enhances the proliferation and chemotactic migration abilities of HER2-CAR T cells, improving their tumor-specific targeting and immune response potential.

### Co-expression of CXCR5 and CCR6 enhances the anti-tumor activity of HER2 CAR T cells in vivo

**Fig. 4:**
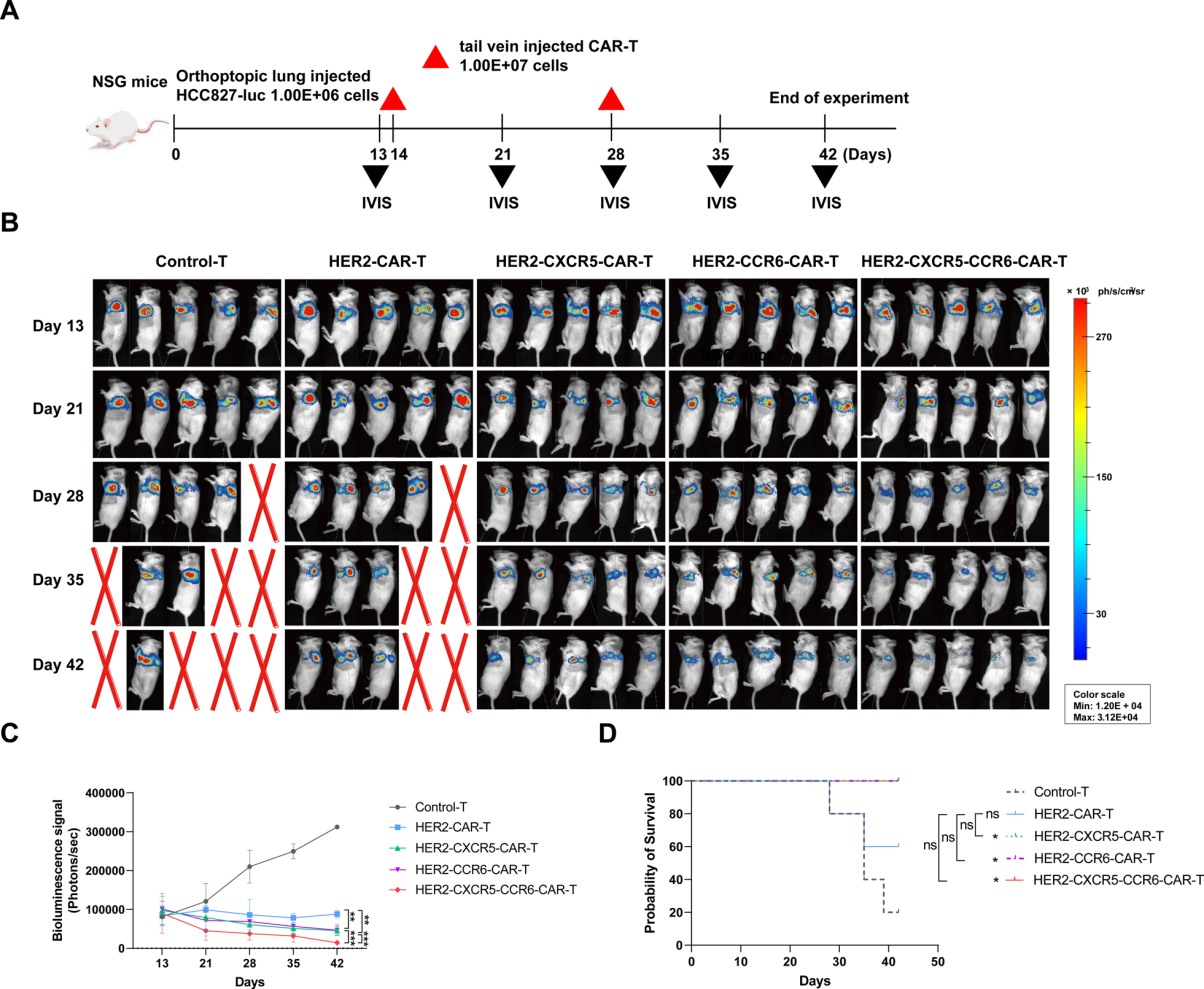
Anti-tumor effects of HER2-T cells can be improved by CXCR5 and CCR6 in vivo. **A** An orthotopic (lung) tumor model was established by HCC827-Luc cells, and after 14 days, tumor-bearing mice were treated with Control-T, HER2-CAR T, HER2-CXCR5-CAR T, HER2-CCR6-CAR T, or HER2-CXCR5-CCR6-CAR T cells. Tumor growth and T cell proliferation were monitored (n = 5 mice per group). **B** Bioluminescence images of tumor-bearing mice treated with Control-T, HER2-CAR T, HER2-CXCR5-CAR T, HER2-CCR6-CAR T, or HER2-CXCR5-CCR6-CAR T cells (n = 5 mice per group). **C** Quantitative analysis of bioluminescence signals. **D** Kaplan–Meier survival analysis. Log-rank test and Two-way ANOVA was conducted for statistical analysis. *P < 0.05, **P < 0.01, ***P < 0.001

To evaluate the in vivo anti-tumor effects of HER2 CAR T cells co-expressing or singly expressing CXCR5 and CCR6, an orthotopic HCC827-Luciferase (intra-lung) tumor-bearing xenograft mouse model was established. Upon achieving a bioluminescence photon flux of 80,000 photons per second (p/s), as detected by the in vivo optical imaging system, HCC827-Luc-bearing mice were randomly assigned to five treatment groups: Control-T, HER2-CAR T, HER2-CXCR5-CAR T, HER2-CCR6-CAR T, and HER2-CXCR5-CCR6-CAR T cells (Fig. 4A). These five groups received different tail-vein transfusions of highly positive FACS-sorted CAR T cells.

The mice treated with HER2-CAR T cells, either co-expressing or singly expressing CXCR5 and CCR6, exhibited significantly improved long-term tumor control compared to those in the Control-T and HER2-CAR T groups. Both the CXCR5 and CCR6 single-expression groups showed a notable enhancement in anti-tumor effects over the HER2-CAR T group, with no significant difference between the two single-expression groups (final tumor bioluminescence photon flux: 45,520 ± 10,305.68 p/s for CXCR5 and 46,760 ± 13,614.07 p/s for CCR6). The HER2-CXCR5-CCR6-CAR T group demonstrated the most effective control over orthotopic HCC827-Luciferase tumors, with a final tumor bioluminescence photon flux of 14,600 ± 5,313.67 p/s, highlighting the synergistic benefits of CXCR5 and CCR6 co-expression (Fig. 4B, C).

In terms of survival, the Control-T and HER2-CAR T groups showed varying degrees of mortality around day 28. In contrast, all mice in the treatment groups with HER2 CAR T cells expressing either CXCR5 or CCR6 singly, or co-expressing both, survived until the end of the experiment. The survival rate of HER2-CXCR5-CAR T group or HER2-CCR6-CAR T group or HER2-CXCR5-CCR6-CAR T group was significantly higher than that of Control-T group (p < 0.05) (Fig. 4D). These findings indicate that Co-expression of CXCR5 and CCR6 enhanced the antitumor efficacy and survival of HER2 CAR T cells in vivo, underscoring the potential of this strategy to improve CAR T therapy for solid tumors.

We further evaluated the in vivo safety of HER2-CXCR5-CCR6-CAR T cells and showed no treatment-related lesions on histological examination of vital organs such as the heart and live (Fig. S6A). And there were no behavioral or weight changes indicative of systemic toxicity (Fig. S6B).

### Co-expression of CXCR5 and CCR6 enhances the in vivo survival and expansion of HER2 CAR T cells

The survival and proliferation of HER2 CAR T cells were monitored on days 21 and 42. The data showed that the mice treated with HER2-CAR T cells co-expressing or singly expressing CXCR5 and CCR6 exhibited a significant increase in the proportion of CD3 + CAR T cells in the peripheral blood compared to those in the HER2-CAR T and Control-T cell treatment groups (Fig. S7A, B). Notably, by day 42, the group co-expressing CXCR5 and CCR6 presented nearly 57.2% CD3 + CAR T cells, whereas the single-expression groups for CXCR5 and CCR6 presented 35.5% and 38.8%, respectively. In contrast, the HER2-CAR T and Control-T cell groups showed no significant changes, with CD3 + CAR T cells remaining below 10% (Fig. S7B).

**Fig. 5:**
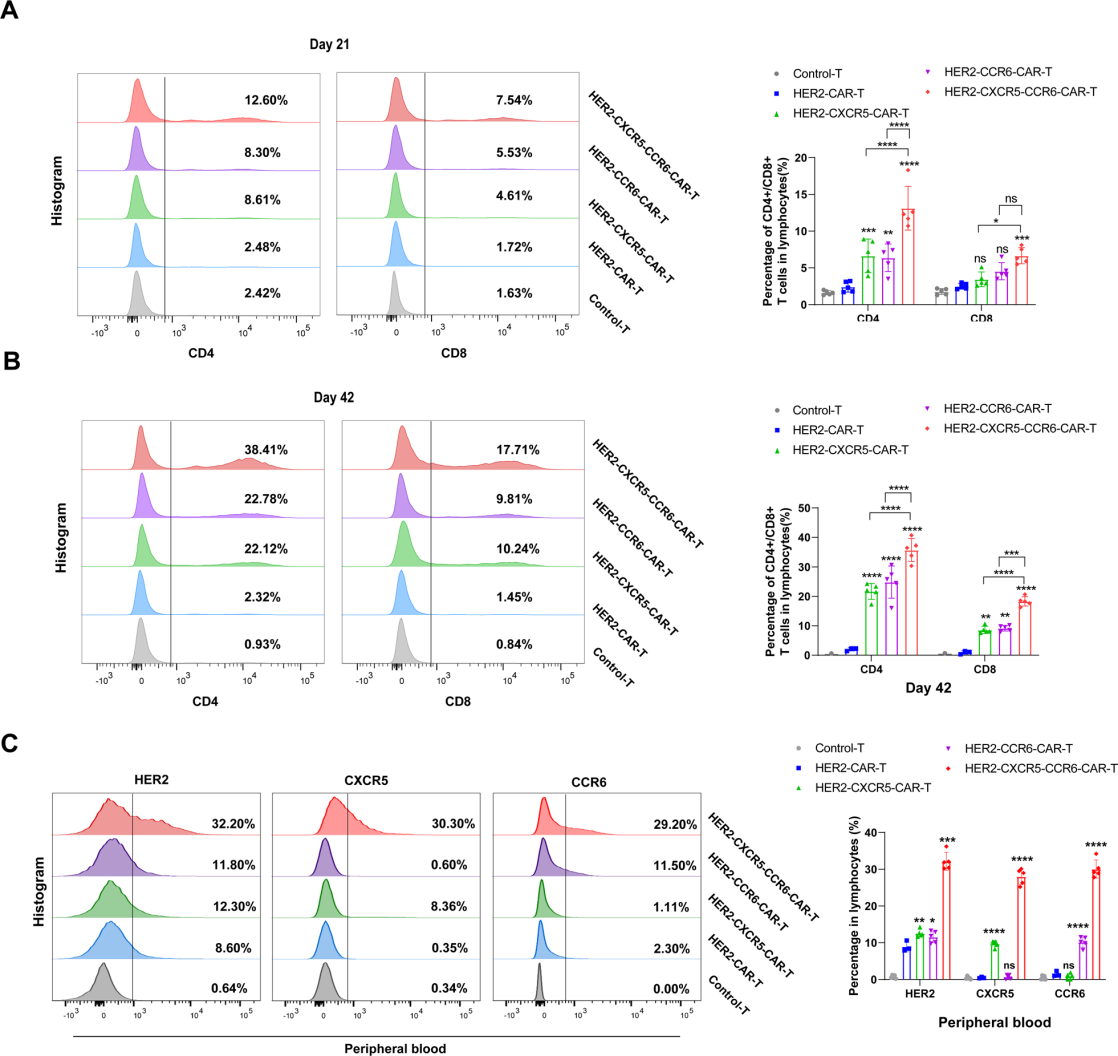
Co-expression of CXCR5 and CCR6 enhanced the expansion and migration of HER2 CAR-T cells in peripheral blood. **A** CD4 + CAR T and CD8 + CAR T cell percentages in peripheral blood lymphocytes detected by flow cytometry on day 21. **B** CD4 + CAR T and CD8 + CAR T cell percentages in peripheral blood lymphocytes detected by flow cytometry on day 42. **C** Expression of HER2-CAR, CXCR5, and CCR6 in peripheral blood T cells measured at the end of the assay (day 42). Unpaired t-test was conducted for statistical analysis. *P < 0.05, **P < 0.01, ***P < 0.001, ****P < 0.0001, ns, no statistical significance

Further analysis showed that on day 21, the proportion of CD4 + and CD8 + CAR T cells in the peripheral blood was increased in the CAR T cells expressing CXCR5 and human CCR6 either alone or in combination, but only the proportion of CXCR5 and CCR6 co-expressing CD8 + CAR T cells was significantly increased in the HER2-CAR T cells (Fig. 5A). Although HER2-CAR T cells expressing CXCR5 or CCR6 alone also showed an increase, this was not significantly different from the HER2-CAR T and Control-T treatment groups (Fig. 5A). By day 42, the HER2-CXCR5-CCR6-CAR T cell treatment group had CD4 + CAR T and CD8 + CAR T cell proportions of 38.4% and 17.7%, respectively, which were significantly higher than those in all other treatment groups (Fig. 5B). Interestingly, HER2-CAR T cells expressing either CXCR5 or CCR6 alone also showed an increase in CD4 + CAR T and CD8 + CAR T cell proportions, reaching 22.1%/10.2% and 22.8%/9.8%, respectively, while the HER2-CAR T and Control-T cell groups remained unchanged at below 3.0% (Fig. 5B).

At the experimental endpoint, the measurement of HER2 CAR-positive cell proportions in the mouse peripheral blood revealed that the HER2-CXCR5-CCR6-CAR T cells maintained a HER2 CAR positivity rate of 32.2%, with CXCR5 and CCR6 expression levels of 30.3% and 29.2%, respectively. In comparison, the HER2 CAR positivity rates for the single-expression CXCR5 or CCR6 groups and the HER2-CAR T group were 11.8% and 12.3%, respectively (Fig. 5C). These findings demonstrate that the co-expression of CXCR5 and CCR6 significantly enhances the survival and expansion of HER2-CAR T cells in vivo, compared to HER2-CAR T cells or those singly expressing CXCR5 or CCR6.

### Co-expression of CXCR5 and CCR6 enhances the in vivo tumor infiltration of HER2 CAR T cells

**Fig. 6:**
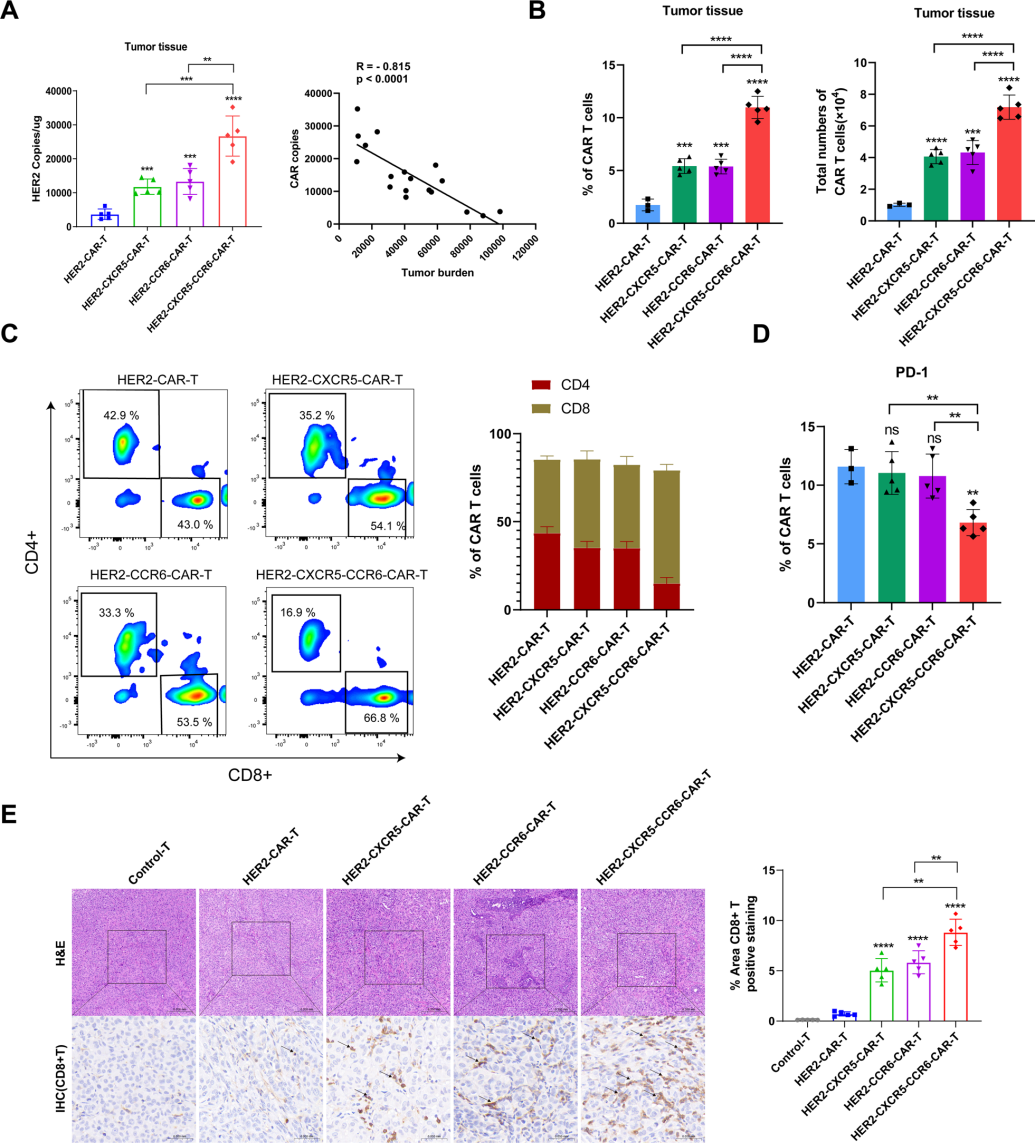
Co-expression of CXCR5 and CCR6 enhances the in vivo tumor infiltration of HER2 CAR T cells. **A** On day 42, the tumor tissues of the mice were removed, real-time fluorescence quantitative PCR was used to detect the HER2-CAR copy number in the genomic DNA, Pearson correlation analysis of CAR copy number and tumor burden. **B** The percentage and total number of CAR T cells infiltrating the tumor tissues (n = 5 mice/group). **C** CD8 and CD4 composition in CAR T cells. **D** The positive rate of the exhaustion marker PD1 in tumor tissues. **E** The other part of the tumor tissue was fixed with formalin and embedded in paraffin, after which CD8 + CAR T cell infiltration was detected by IHC. Unpaired t-test and Pearson correlation analysis was conducted for statistical analysis. **P < 0.01, ***P < 0.001, ****P < 0.0001

To mechanistically interrogate enhanced therapeutic efficacy, we conducted an analysis of HER2 CAR T cell infiltration within the tumor tissue. qPCR data revealed that the HER2-CXCR5-CCR6-CAR T cells displayed the highest copy number of HER2 cells in the tumor tissue (Fig. 6A), with the groups expressing either CXCR5 or CCR6 alone showing intermediate levels, both significantly exceeding those of the HER2-CAR T and Control-T group. And higher HER2-CAR copy numbers in tumor tissue inversely correlated with terminal tumor burden (Pearson R = −0.815, P < 0.001; Fig. 6A). Furthermore, comprehensive flow cytometry analysis of tumor-infiltrating lymphocytes (TILs) harvested at the experimental endpoint (Day 42) revealed that HER2-CXCR5-CCR6-CAR T treatment significantly enhanced both the frequency and total number of infiltrating CAR T cells within tumor tissues compared to HER2-CXCR5-CAR T and HER2-CCR6-CAR T treatments (Fig. 6B and Fig. S8A-C). Subset analysis demonstrated a significantly higher enrichment of CD8 + CAR T cells within the tumor tissue of the HER2-CXCR5-CCR6-CAR T group. Conversely, the HER2-CXCR5-CAR T and HER2-CCR6-CAR T groups exhibited a predominance of CD4 + CAR T cells (Fig. 6C and Fig. S8D). Additionally, PD-1 expression on tumor-infiltrating CAR T cells was significantly lower in the HER2-CXCR5-CCR6-CAR T group than in the other treatment groups, indicative of reduced T cell exhaustion (Fig. 6D). IHC experiments were conducted on the tumor tissue to assess the infiltration of CD4 + and CD8 + CAR T cells across different treatment groups. Both the co-expression and single expression of CXCR5 or CCR6 in HER2-CAR T cells resulted in a markedly higher positive staining for CD4 + and CD8 + CAR T cells in the tumor tissue compared to those in the HER2-CAR T cell and Control-T treatment groups (Fig. 6E and Fig. S9A). Detailed statistical analyses of the CD4 + and CD8 + positive rates demonstrated that the HER2-CXCR5-CCR6-CAR T group had infiltration rates of 5.4% for CD4 + CAR T and 8.8% for CD8 + CAR T cells in the tumor tissue. The groups expressing CXCR5 or CCR6 alone exhibited infiltration rates of 2.1%/3.8% for CD4 + and 5.0%/5.8% for CD8 + CAR T cells. The data from all the treatment groups, either co- or singly expressing CXCR5 or CCR6, were significantly higher than those from the HER2-CAR T group, which had CD4 + CAR T cell infiltration at 0.2% and CD8 + CAR T cell infiltration at 0.7% (Fig. 6E and Fig. S9B). This suggests that the co-expression of CXCR5 and CCR6 significantly enhances the ability of HER2 CAR T cells to infiltrate tumors in vivo.

## Discussion

The heterogeneity of solid tumors and the complexity of the TME present significant challenges to the effective infiltration and functionality of CAR T cells [33]. Although the regional delivery of CAR T cells has made strides in addressing deep tissue penetration and minimizing systemic side effects in solid tumor therapies, it remains limited in terms of overall targeting and therapeutic efficacy [34, 35]. A promising alternative approach combines the intrinsic mechanisms of T cells with chemotactic guidance, aiming to improve T cell migration and adaptation within the TME. Notably, CAR T cell therapies that co-express chemokine receptors have shown great promise in enhancing T cell migration and infiltration into tumors [36]. The high specificity of chemokine receptor–ligand pairs ensures more precise targeting, preventing unintended off-target effects and adverse reactions due to redundant chemokine receptor–ligand interactions [37].

In this study, we identified the elevated expression of chemokines CXCL13 and CCL20 in the TME of NSCLC, based on an analysis of a highly specific set of six orphan chemokine receptor–ligand pairs. By modifying CAR T cells to co-express the specifically paired receptors CXCR5 and CCR6, we demonstrated that HER2-CXCR5-CCR6-CAR T cells could respond to the CXCL13 and CCL20 chemokines present in the TME, thereby enhancing their ability to migrate and home effectively to solid tumor sites. Importantly, we found that HER2-CXCR5-CCR6-CAR T cells, in addition to demonstrating migration, homing, and anti-tumor effects, also exhibited significant expansion and survival advantages, as confirmed by both in vitro and in vivo experiments. Critically, our data reveal a strong positive correlation between sustained CAR T cell persistence and tumor control efficacy. As evidenced by longitudinal monitoring (Figs. 4C and 5B), the HER2-CXCR5-CCR6-CAR T group maintained significantly higher peripheral CAR T cell frequencies (32.2% vs. ≤ 12.3% in single-receptor groups at Day 42), which directly paralleled superior tumor suppression (14,600 ± 5,313.67 p/s vs. ≥ 45,520 p/s in controls). This durable persistence correlated with enhanced intratumoral CAR T infiltration (Fig. 6A, B) and reduced exhaustion (Fig. 6D), suggesting that prolonged CAR T activity is a key determinant of long-term antitumor responses. Such durability may be attributed to synergistic chemokine-driven survival signals within the TME, providing a functional reservoir for continuous tumor surveillance.

CXCL13 in the TME can attract tumor-specific T cells, promoting their activation and proliferation to enhance immune responses [14, 26]. Similarly, CCL20 modulates macrophage polarization and influences the immune status of the TME [25]. We hypothesize that the enhanced proliferation and survival of HER2-CXCR5-CCR6-CAR T cells are driven by the chemotactic actions of the CXCL13–CXCR5 and CCL20–CCR6 axes. These actions are synergistically enhanced by the immune-boosting effects of the CXCL13–CXCR5 axis and the immunomodulatory effects of the CCL20–CCR6 axis within the tumor. This synergistic enhancement of anti-tumor efficacy was validated in our in vitro assays and in vivo models, wherein we observed improved cytotoxicity, tumor infiltration, cytokine secretion, and anti-tumor effects. Collectively, our findings suggest that the co-expression of CXCR5 and CCR6 confers a distinct advantage, providing synergistic anti-tumor effects over the single expression of either receptor in HER2-CAR T cells.

**Fig. 7:**
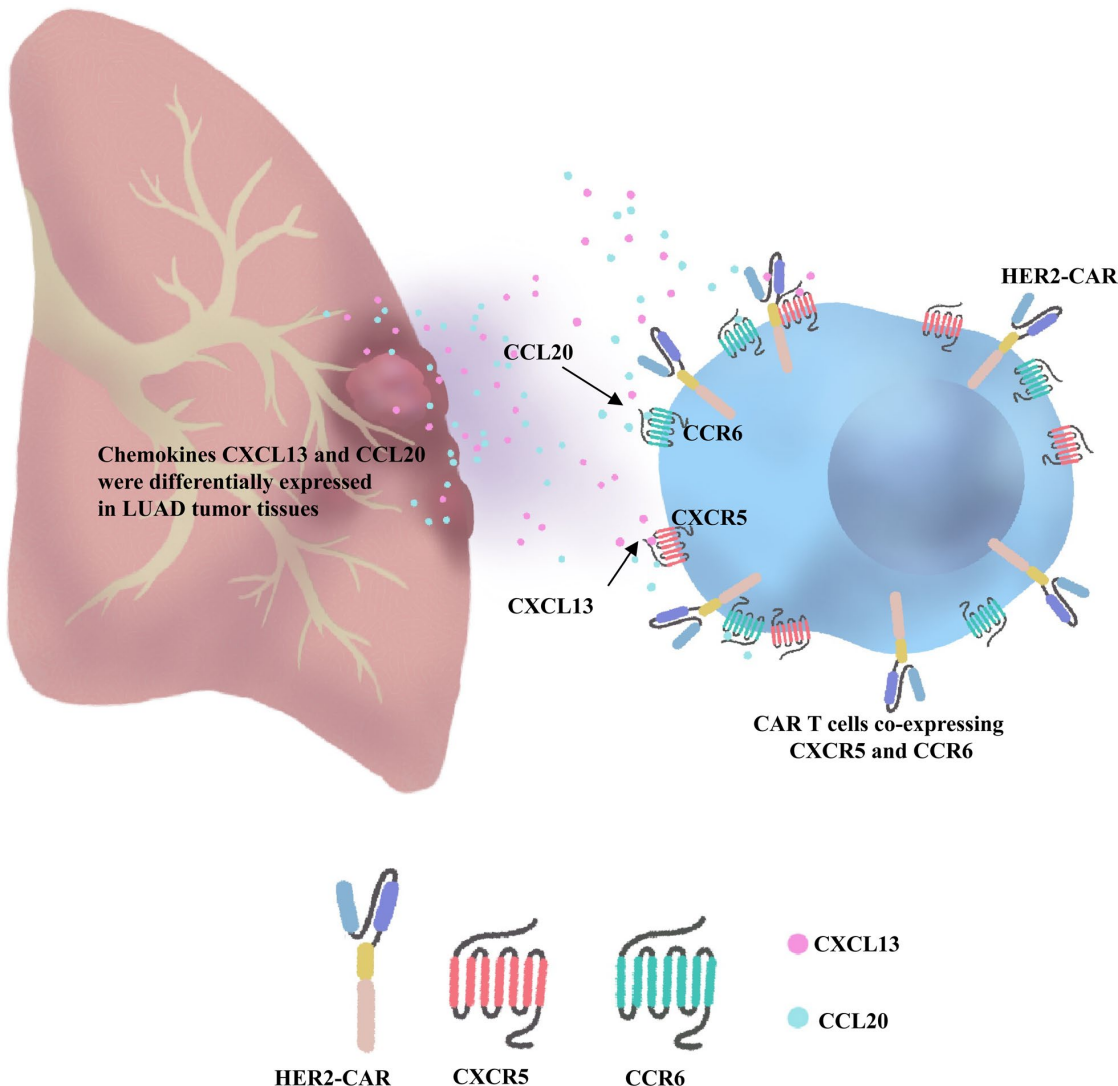
Co-expression of CXCR5 and CCR6 enhances the homing and killing mechanisms of HER2-CAR T cells. HER2-CAR-CXCR5-CCR6-T cells targeting chemokines CXCL13 and CCL20 exhibit more abundant solid lung tumor cell infiltration, more extensive proliferation, and stronger anti-tumor effects

Notably, HER2-CXCR5-CCR6-CAR T cells demonstrated significantly enhanced tumor infiltration compared to conventional HER2-CAR T cells, attributable to their triple-targeting capacity for tumor sites co-expressing HER2, CXCL13, and CCL20. This multi-receptor strategy functions as a molecular navigation system (Fig. 7), effectively minimizing off-tumor toxicity risks associated with single-antigen targeting while addressing key challenges in solid tumor therapy. The approach further enables personalized intervention through tumor microenvironment (TME) chemokine profiling, allowing tailored CAR-T designs aligned with patient-specific expression patterns.

Our analysis of 483 LUAD samples and 347 normal tissue samples from the TCGA and GTEx databases revealed that the expression levels of CXCL13 and CCL20 in lung adenocarcinoma tissues were significantly higher compared to normal lung tissues (Fig. 1A). Additionally, we confirmed these findings in the tissue samples of 6 lung adenocarcinoma patients (Fig. 1B). Therefore, the considerations for translation include the presence of clinically significant differences in chemokine expression among non-small cell lung cancer patients, this inter-patient variability in chemokine axis expression necessitates pretreatment TME screening for optimal patient stratification. Regarding the safety concerns regarding the tumor extrinsic toxicity risk caused by HER2 expression in normal tissues, we conducted histological examinations of important organs such as the mouse heart and found no treatment-related lesions (Fig. S6A). Additionally, no behavioral or weight changes indicative of systemic toxicity were observed (Fig. S6B). Regarding the complexity of the production of dual-receptor CAR-T cells, although the transduction efficiency of the HER2-CXCR5-CCR6-CAR in vitro exceeded 60% (Fig. 2B), the expansion of production scale still poses challenges. This requires future optimization of lentiviral vector engineering to achieve clinical translation.

Despite the promising results, the clinical application of this multi-receptor targeting strategy requires further validation. Given the heterogeneity and complexity of tumors, it is unlikely that a single treatment approach will be effective across all types of lung cancer. Future research should explore the applicability of this strategy across different lung cancer subtypes and evaluate its combined effects with other treatments, such as chemotherapy, radiotherapy, and immune checkpoint inhibitors. By considering these factors, we can develop a more comprehensive and effective treatment strategy for patients with lung cancer.

## Supplementary Information

Supplementary material 1.

Supplementary material 2.

Supplementary material 3.

Supplementary material 4.

Supplementary material 5.

Supplementary material 6.

Supplementary material 7.

Supplementary material 8.

Supplementary material 9.

Supplementary material 10.

Supplementary material 11.

## Data Availability

All data are available in the main text or the Supplementary Materials.
