# Supplementary material for: Enhanced homing and efficacy of HER2-CAR T cells via CXCR5/CCR6 co-expression for HER2-positive NSCLC
Source: J Transl Med. 2025 Aug 5;23:863. doi: 10.1186/s12967-025-06866-9 (PMC12326854; doi:10.1186/s12967-025-06866-9)
Supplement: Supplementary file 1 — Supplementary material 1. [file 12967_2025_6866_MOESM1_ESM.docx]

Supplementary Material

**Enhanced Homing and Efficacy of HER2-CAR T Cells via CXCR5/CCR6 Co-Expression for HER2-Positive NSCLC**

Xiaoyuan Hu ^1#^, Chunlei Ge^1#^, Caixiu Huang^1#^, Dan He^1^, Xiaoxuan Yao^1^, Jiaxing Cheng^1^, Jiyin Guo^1^, Ke Li^1^, Yunshan Ye^1^, Li Li^1^, Jianchuan Xia^2^, Tao Li^1*^, Hong Yao^1*^.

**AFFILIATIONS:**

1 Cancer Biotherapy Center& Cancer Research Institute, The Third Affiliated Hospital of Kunming Medical University, Yunnan Cancer Hospital, Peking University Cancer Hospital Yunnan, Kunming, China.

2 State Key Laboratory of Oncology in South China, Guangdong Provincial Clinical Research Center for Cancer, Guangdong Key Laboratory of Nasopharyngeal Carcinoma Diagnosis and Therapy, Sun Yat-sen University Cancer Center, Guangzhou, Guangdong, 510060, P. R. China.

***Corresponding Author:** Hong Yao, Cancer Biotherapy Center& Cancer Research Institute, Yunnan Cancer Hospital, The Third Affiliated Hospital of Kunming Medical University, Peking University Cancer Hospital Yunnan, Kunming, 650106, China, E-mail address: [yaohong20055@hotmail.com](mailto:yaohong20055@hotmail.com);

Tao Li, Cancer Biotherapy Center& Cancer Research Institute, Yunnan Cancer Hospital, The Third Affiliated Hospital of Kunming Medical University, Peking University Cancer Hospital Yunnan, Kunming, 650106, China, Kunming, 650106, China, E-mail address: [litaove@163.com](mailto:litaove@163.com);

# Xiaoyuan Hu, Chunlei Ge and Caixiu Huang contributed equally to this manuscript.

**Supplementary Figure 1. Vector map of pCDH-CAR.**

1. Vector map of pCDH-HER2-CAR.
2. Vector map of pCDH-HER2-CXCR5-CAR.
3. Vector map of pCDH-HER2-CCR6-CAR.
4. Vector map of pCDH-HER2-CXCR5-CCR6-CAR.

**Supplementary Figure 2. Expression of chemokines in lung adenocarcinoma (LUAD).**

A. Schematic representation of chemokine and their receptor binding.

B. The expression of CCL25, CXCL13, CXCL16 and CXCL12 in patients with LUAD using the online tool of GEPIA.

C．Kaplan-Meier survival analysis for CXCL13 and CCL20 in LUAD tumors, performed using the GEPIA tool.

**Supplementary Figure 3. HER2 and Chemokine CXCL13, CCL20 expression profile in LUAD tumors.**

The mRNA expression differences of HER2, CXCL13 and CCL20 in carcinoma and adjacent tissues in six LUAD tumor samples was detected by qRT-PCR.

**Supplementary Figure 4. HER2 and Chemokine CXCL13, CCL20 expression profile in LUAD cell lines.**

1. The relative expression levels of HER2, CXCL13 and CCL20 proteins in 6 LUAD cell lines and 1 normal bronchopulmonary epithelial cell line BEAS-2B.

B.The expression levels of CXCL13 and CCL20 in the six LUAD cell lines and one normal bronchopulmonary epithelial cell line BEAS-2B cell culture supernatant were detected by ELISA.

C. The mRNA expression of HER2, CXCL13, and CCL20 in six LUAD cell lines and one normal bronchopulmonary epithelial cell line BEAS-2B was detected by qRT-PCR.

D. The expression of HER2 on the surface of HCC827 and BEAS-2B cells was detected by flow cytometry.

E. The expression levels of HER2 protein in HER2-CAR-T cells before and after sorting were detected by flow cytometry.

**Supplementary Figure 5.** **The representative flow cytometry gating strategies and validation of Figure 2C**

A. FSC-A vs. SSC-A (debris exclusion) → Live HER2-CXCR5-CCR6-CAR-T cells.

B. CD69 expression histograms for ISO, control, Incubate with 1 × 10^3^ target cells.

**Supplementary Figure 6. In vivo safety assessment of HER2-CXCR5-CCR6-CAR-T cells**

A. HE staining of heart and liver of NSG mice from different CAR-T cell treatment groups.

B. Body weight changes of NSG mice in different CAR-T cell treatment groups were detected.

**Supplementary Figure 7. CXCR5 and CCR6 enhanced proliferation and migration and infiltration to tumors of HER2-CAR-T cells *in vivo*.**

A-B. CD3+ CAR-T cells percentage in peripheral blood lymphocytes was detected by flow cytometry on day 21 and day 42.

**Supplementary Figure 8. The representative flow cytometry gating strategies and validation of Figure 6D-E**

A. FSC-A vs. SSC-A (debris exclusion) →Single cells.

B. The proportion of CD3+ T cells in single cells.

C. The proportion of CAR-T cells in CD3+ T cells.

D. The proportion of CD4 and CD8+ T cells in CAR-T cells.

**Supplementary Figure 9. Tumor tissues were fixed with formalin, embedded in paraffin, and used for IHC to detect the infiltration of CD4+ CAR-T cells.**
