# Supplementary material for: Enhanced homing and efficacy of HER2-CAR T cells via CXCR5/CCR6 co-expression for HER2-positive NSCLC
Source: J Transl Med. 2025 Aug 5;23:863. doi: 10.1186/s12967-025-06866-9 (PMC12326854; doi:10.1186/s12967-025-06866-9)
Supplement: Supplementary file 2 — Supplementary material 2. [file 12967_2025_6866_MOESM2_ESM.docx]

**Supplementary Table 1.** The expression profile of the gray value of chemokine protein levels in six NSCLC cell lines and a normal bronchial epithelial cell line BEAS-2B.

| **Gray value** | **CXCL13/GAPDH** | **CCL20/GAPDH** |
| --- | --- | --- |
| **BEAS-2B** | **0.232±0.034** | **0.903±0.0573** |
| **PC-9** | **0.682±0.033** | **1.293±0.021** |
| **A549** | **0.513±0.033** | **2.517±0.041** |
| **H1734** | **0.733±0.042** | **2.627±0.041** |
| **H1975** | **1.063±0.045** | **3.400±0.059** |
| **H1299** | **1.687±0.209** | **1.610±0.054** |
| **HCC827** | **1.710±0.124** | **2.687±0.168** |

**Supplementary Table 2.** The expression profile of chemokine mRNA levels in six NSCLC cell lines and a normal bronchial epithelial cell line BEAS-2B.

| **mRNA** | **CXCL13/GAPDH** | **CCL20/GAPDH** |
| --- | --- | --- |
| **BEAS-2B** | **0.00083±0.00026** | **0.00119±0.00013** |
| **PC-9** | **0.00580±0.00156** | **0.01101±0.00051** |
| **A549** | **0.00627±0.00252** | **0.08246±0.01313** |
| **H1734** | **0.00696±0.00157** | **0.05805±0.00038** |
| **H1975** | **0.00394±0.00088** | **1.32414±0.14556** |
| **H1299** | **0.01514±0.00651** | **0.03267±0.00572** |
| **HCC827** | **0.01514±0.00206** | **0.75742±0.12996** |

Supplementary Table 3. Statistical analysis of all E:T ratios in Figure 2D

| E: T Ratio | Comparison Group | Cytotoxicity (% Killing) | P value |
| --- | --- | --- | --- |
| 1: 1 | HER2-CAR-T vs HER2-CXCR5-CAR-T | 12.97±1.14 vs 13.18±1.35 | ns |
|  | HER2-CAR-T vs HER2-CCR6-CAR-T | 12.97±1.14 vs 13.16±1.01 | ns |
|  | HER2-CXCR5-CAR-T vs HER2-CXCR5-CCR6-CAR-T | 13.18±1.35 vs 12.67±1.36 | ns |
|  | HER2-CCR6-CAR-T vs HER2-CXCR5-CCR6-CAR-T | 13.16±1.01 vs 12.67±1.36 | ns |
| 5: 1 | HER2-CAR-T vs HER2-CXCR5-CAR-T | 14.37±0.63 vs 15.51±0.52 | ns |
|  | HER2-CAR-T vs HER2-CCR6-CAR-T | 14.37±0.63 vs 15.24±0.81 | ns |
|  | HER2-CXCR5-CAR-T vs HER2-CXCR5-CCR6-CAR-T | 15.51±0.52 vs 16.5±0.78 | ns |
|  | HER2-CCR6-CAR-T vs HER2-CXCR5-CCR6-CAR-T | 15.24±0.81 vs 16.5±0.78 | ns |
| 10: 1 | HER2-CAR-T vs HER2-CXCR5-CAR-T | 18.23±0.95 vs 19.48±0.46 | ns |
|  | HER2-CAR-T vs HER2-CCR6-CAR-T | 18.23±0.95 vs 18.93±0.49 | ns |
|  | HER2-CXCR5-CAR-T vs HER2-CXCR5-CCR6-CAR-T | 19.48±0.46 vs 22.33±0.86 | ns |
|  | HER2-CCR6-CAR-T vs HER2-CXCR5-CCR6-CAR-T | 18.93±0.49 vs 22.33±0.86 | p<0.05* |
| 20: 1 | HER2-CAR-T vs HER2-CXCR5-CAR-T | 21.67±0.45 vs 30.50±0.78 | p<0.0001**** |
|  | HER2-CAR-T vs HER2-CCR6-CAR-T | 21.67±0.45 vs 27.78±1.48 | p<0.0001**** |
|  | HER2-CXCR5-CAR-T vs HER2-CXCR5-CCR6-CAR-T | 30.50±0.78 vs 38.82±2.24 | p<0.0001**** |
|  | HER2-CCR6-CAR-T vs HER2-CXCR5-CCR6-CAR-T | 27.78±1.48 vs 38.82±2.24 | p<0.0001**** |

ns, not statistically significant; *, p<0.05; ****, p<0.0001
