# Supplementary figures and images for: Enhanced homing and efficacy of HER2-CAR T cells via CXCR5/CCR6 co-expression for HER2-positive NSCLC
Source: J Transl Med. 2025 Aug 5;23:863. doi: 10.1186/s12967-025-06866-9 (PMC12326854; doi:10.1186/s12967-025-06866-9)

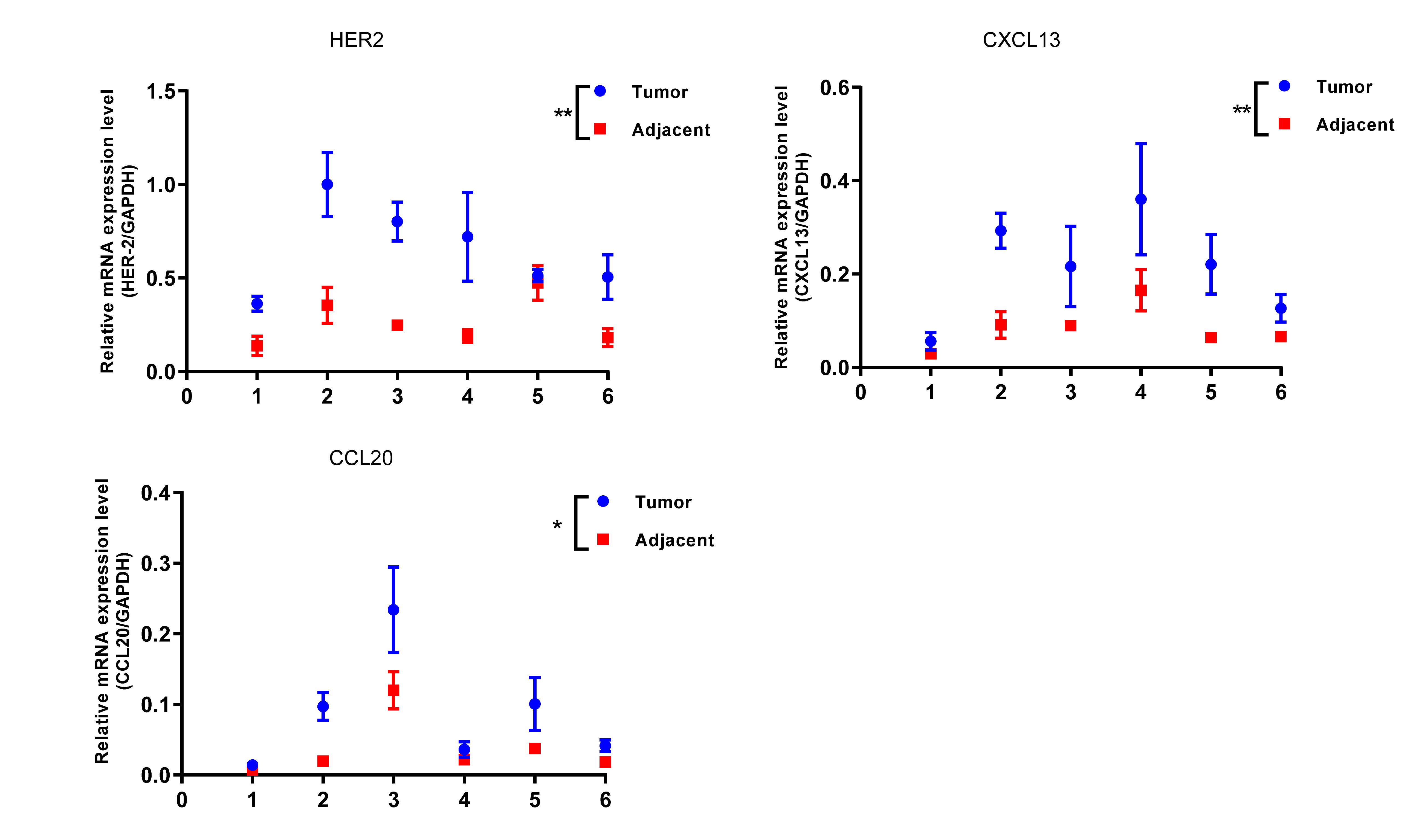

Supplement: Supplementary file 5 — Supplementary material 5. [file 12967_2025_6866_MOESM5_ESM.tif]

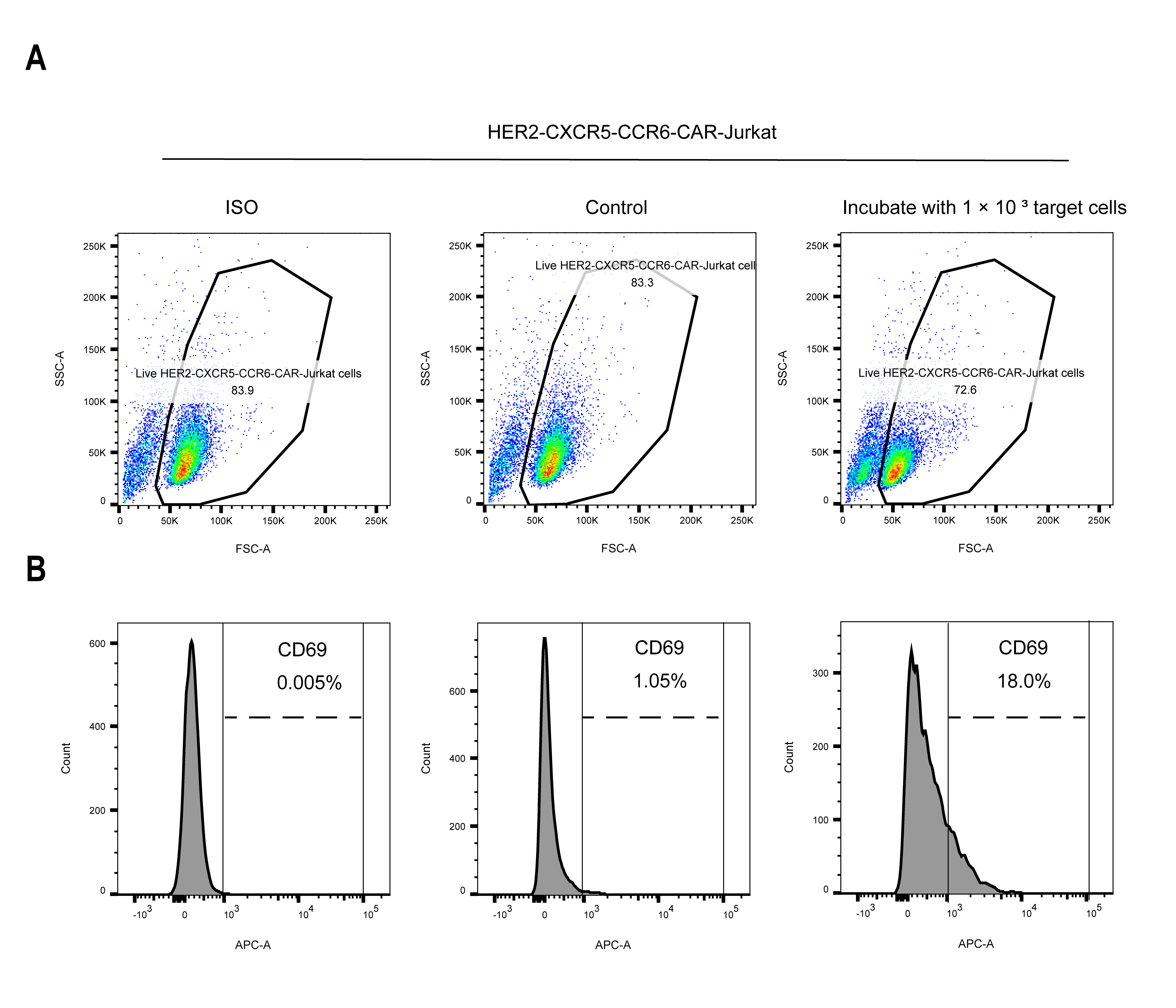

Supplement: Supplementary file 7 — Supplementary material 7. [file 12967_2025_6866_MOESM7_ESM.tif]
